# [Retracted] Guggulsterone inhibits human cholangiocarcinoma Sk-ChA-1 and Mz-ChA-1 cell growth by inducing caspase-dependent apoptosis and downregulation of survivin and Bcl-2 expression

**DOI:** 10.3892/ol.2026.15448

**Published:** 2026-01-07

**Authors:** Fei Zhong, Jing Yang, Zhu-Ting Tong, Liu-Liu Chen, Lu-Lu Fan, Fang Wang, Xia-Li Zha, Jun Li

Oncol Lett 10: 1416–1422, 2015; DOI: 10.3892/ol.2015.3391

Following the publication of this paper, it was drawn to the Editor's attention by a concerned reader that, regarding the western blot data shown in Fig. 5, the Bcl2 / Sk-ChA-1 gel slice looked remarkably similar to the Survivin / Mz-ChA-1 gel slice if one of the two were flipped vertically, and lanes 1–3 of the β-actin / Sk-ChA-1 panel looked strikingly similar to lanes 2–4 of the β-actin / Mz-ChA-1 panel. Upon performing an independent analysis of the data in this paper in the Editorial Office, it also came to light that western blot data in Fig. 5A and certain of the DNA ladder electrophoresis data in Fig. 3B had apparently already been published in other articles that were written by different authors at different research institutes.

Owing to the fact that the contentious data in the above article had already apparently been published prior to its submission to *Oncology Letters*, the Editor has decided that this paper should be retracted from the Journal. AThe authors were asked for an explanation to account for these concerns, but the Editorial Office did not receive a reply. The Editor apologizes to the readership for any inconvenience caused.

